# In Vitro Hb Production in B-thalassemia Patients Is Not a Predictor of Clinical Responsiveness to Hydroxyurea

**Published:** 2017-07

**Authors:** Mohammad Reza MAHDAVI, Farzin POURFARZAD, Mehrnoush KOSARYAN, Mohammad Taghi AKBARI

**Affiliations:** Dept. of Medical Genetics, School of Medical Sciences, Tarbiat Modares University, Tehran, Iran

**Keywords:** Hydroxyurea, Thalassemia, Hemoglobin

## Abstract

**Background:**

The hematologic response to hydroxyurea (HU) is varied among β-thalassemia (BT) patients. The BCL11A and SOX6 genes are involved in response to HU. This study aimed to investigate the in-vitro responsiveness of HU among BT major patients homozygote for IVSII-1G>A mutation and XmnI single nucleotide polymorphism (SNP) in order to find whether the in-vitro Hb concentration is a predictor of clinical (HU) responsiveness.

**Methods:**

In this case-control study, twenty BT patients homozygote for IVSII-1G>A mutation and XmnI SNP from Thalassemia Research Center, Sari, Iran in 2015 were selected and categorized into two groups of 10 Responder (R) and 10 Non-Responder (NR) according to their clinical HU response. Ten healthy individuals as a control group were also selected. Hematopoietic erythroid progenitors were expanded from peripheral blood. Hb concentration was measured using photometry method. The flow cytometry and real-time PCR methods were applied for the analysis of cell surface markers (CD71 and CD235a) and gene expression (BCL11A and SOX6), respectively.

**Results:**

R and NR groups produced higher amount of Basic Hb than C group in cell culture medium at day 14 (*P*<0.05). After HU treatment, in R group, Hb levels was significantly elevated in comparison to NR and C group (*P*<0.05). BCL11A expression was decreased after exposure to HU in all groups while SOX6 expression was only down-regulated in C group, and its expression was increased in R and NR groups after HU treatment.

**Conclusion:**

Since different factors including wide networks of intracellular factors and individual differences between patients can affect response to HU in patients, the increasing Hemoglobin on culture medium alone cannot predict clinical responsiveness to that drug.

## Introduction

Beta thalassemia (BT) is one of the most frequent hemoglobin disorder characterized by reduced or lack of the β-globin protein production. The lack of β-globin chain production leads to accumulation of free intracellular α-globin chains thought to cause oxidative damage to the red cell membrane and induce apoptosis of erythroid precursors (1). During last two decades, chemical induction of fetal hemoglobin (HbF) ameliorated pathologic symptoms of BT patients and increased their quality of life. In BT, increasing levels of HbF reduces the alpha and beta globin chain imbalance and leads to decreased RBC destruction by ineffective erythropoiesis (2). Hydroxyurea (HU) is a toxic agent used for treatment of myeloproliferative disease (MPD) and approved by FDA for treatment of patients with Sickle Cell Disease (SCD) and it has been widely used for improvement of clinical manifestation in BT (3). HU improves hematologic parameters and causes transfusion independence. The hematologic response to HU is varied among patients. Different genetic backgrounds of patients are supposed to be involved in the variation of response to HU (4, 5).

A substantial decrease on α/non-α chain imbalance such as expression of γ-chains that can bind to extra α–chains (6) and associated α–globin gene deletions that reduce the excess of α-globin chains lead to a less severe form of β-thalassemia (7). Quantitative Trait Loci (QTL) including XmnI, BCL11A, and HBSB1L-cMYB SNPs are responsible for 20%–50% of HbF elevation. The -158 C>T XmnI SNP (rs7482144), is the most important factor involved in HbF elevation located on γ-globin gene promoter (8). Some studies have also mentioned the probable role of IVSII-1G>A mutation (one of the common mutation on β-globin gene) on elevation of HbF in response to HU (9, 10).

BCL11A and SOX6 are two important genes involved in Hb switching process. BCL11A is a major regulator of fetal hemoglobin production. In adult human erythroid precursors, down-regulation of BCL11A expression leads to induction of HbF (11). While a precise role for SOX6 in hemoglobin switching is not well known, SOX6 appears to act as either an inducer or a repressor of HbF production, depending on its interacting proteins and promoter context (12).

The aim of this study was to evaluate in vitro Hb production, BCL11A and SOX6 genes expression among R and NR β-thalassemia patients that were homozygote for IVSII-1G>A mutation and XmnI SNP to predict clinical responsiveness to HU.

## Materials and Methods

### Patients

Around 800 BT patients have been registered at Thalassemia Research Center, Hemoglobinopathy Institute, Boo-Ali Hospital, Sari, Iran, since 1986. ARMS-PCR method for identification of the IVSII-1G>A mutation was carried out on 200 patients and 60 of them were homozygote for the mentioned mutation. Of these 60 cases, 49 had XmnI (+/+) haplotype, and 9 of 49 cases had one of the α-thalassemia deletions (−− Med, α^3.7^, α^4.2^, and ααα^Anti3.7^) and excluded from study.

Finally, in this case-control study, 20 out of 40 BT patients that had the inclusion criteria’s attended the study and were categorized into two groups of 10 R and 10 NR according to their clinical response to HU based on medical records. Members of R group clinically had a good response to HU treatment and were able to maintain hemoglobin level up to 8.5 g/dl (threshold for transfusion) and as a result, they become transfusion independent, while NR patients had poor response to HU treatment with less than 8.5 g/dl and remained dependent on regular blood transfusion. Ten healthy individuals with no hemoglobinopathy – based on hemoglobin (Hb) electrophoresis analysis- were also included as a control (C) group. All members of C group were -/- for XmnI SNP and did not carry α-thalassemia deletions.

This study was approved by Ethics Committee of Mazandaran University of Medical Sciences and all participants signed an informed consent.

### Cell culture

Hematopoietic Erythroid Progenitors (HEP) were cultured from peripheral blood as published (14). Before sampling, R group did not take HU for four weeks and the samples of NR group were taken after 28 d of last transfusion. Forty ml of blood samples were collected by venoject tubes and buffy coats were isolated by centrifugation. Peripheral blood mononuclear cells (PBMC) were isolated by density purification using Percoll (GE Healthcare, Little Chalfont, UK) with density of ϱ1.077.

For expansion phase (Phase 1), cells were cultured at density of 1-2×10^6^ cells/ml in serum-free medium (StemSpan; Stem Cell Technologies, Canada) enriched with lipids (40 ng/ml cholesterol-rich lipid mix; Sigma) and supplemented with erythropoietin (2 u/ml, Sigma), IL-3 (1 ng/m; Sigma), dexamethasone (1 μM; Sigma) and SCF(Stem Cell Factor) (50 ng/ml, Sigma). Cells were expanded until day 7 by partial daily medium changes and addition of mentioned growth factors with keeping cell densities between 1.5–2×10^6^ cells/ml. On day 7, erythroblasts were purified by density purification (Percoll, ϱ1.075) to remove lymphocytes. The cells were recultured in expansion medium without IL-3 (phase 2, proliferation phase) and they were expanded until day 7 by partial daily medium changes and addition of fresh mentioned factors with keeping cell densities between 1.5–2 ×10^6^cells/ml. On day 14 (Phase 3, Differentiation phase), the cells were treated with 100 μM/mL of HU (Sigma, Germany) and the cell culture process was continued until day 16. Hb content, expression of CD71 and CD235a markers, and BCL11A and SOX6 genes expression were evaluated before adding HU in start of third phase of cell culture on day 14 and 48 h after HU treatment, on day 16.

### Optimizing HU concentration in cell culture

**Fig. 1:**
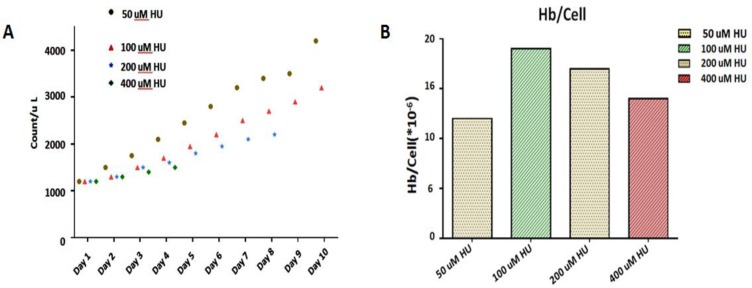
The results of cell count and Hb production on different Hu concentration; A) Cell count per day, B) Hb per cell

To avoid toxic concentration of HU and finding the optimized HU concentration on erythroid progenitors that causes the highest total Hb content, various concentration of HU (50 μM, 100 μM, 200 μM, and 400 μM) were used in cultured HEPs isolated from two healthy donors for 10 d (Fig. 1A).

### Evaluating Erythroid lineage maturation and Quantification of total Hb

Erythroid lineage maturation was assessed using monoclonal antibodies against CD71 (BD, USA) and CD235a (BD, USA) by flow cytometry technique (Partec, Cube 6, Germany) in cell culture (13) on days 12, 14, and 16 of cell culture.

The CFU-E erythroid precursor expresses only CD71 molecule on their surfaces and pure RBC express only CD235a on their surfaces. Double positive cells represent the cell series from pronormoblast to reticulocytes lineage. Hb content was measured using photometry in aliquots of approximately 2×10^6^ cells of the original cultures (14).

### RNA extraction and cDNA synthesis

Total RNA was extracted from the cells using QiagenRNeasy Mini Kit. The isolated RNA was treated with RNase-free DNaseI (EN0525, Thermo scientific) to eliminate possible genomic DNA contamination. The cDNA was synthesized using Prime Script First-Strand cDNA Synthesis Kit (6110B, Takara, Japan).

### Real-time PCR

Quantitative real-time PCR was performed on Rotor gene-6000 (Corbett Australia). For each reaction 12.5 μl of SYBR Premix Ex Taq II master mix (RR820L, Takara, Japan), 10 pmol of each forward and reverse primers, and 2 μl of cDNA were applied in final volume of 25 μl. The PCR program was as follows for all reactions: 1 min at 95 °C followed by 40 cycles of 15 sec at 95 °C and 30 sec at 60 °C and melting curve analysis was performed at the end of PCR-program. The primers were designed spanning intron/exon junctions. Human USP 14 used as a reference gene for normalization and comparative CT method was used for enrichment of specific genes (15) (Table 1).

**Table 1:** Sequences of primers used for evaluation of BCL11A and SOX6 genes expression

**Primer Name**	**Sequence (5’→3′)**
BCL11A/F	GTCTCGCCGCAAGCAAGG
BCL11A/R	GCCGTGGTCTGGTTCATCATC
SOX6/F	CGAGACAACAGCAGCAACTTC
SOX6/R	GAGTCCGCTGGTCATGTGG
USP14/F	AACGCTAAAGGATGATGATTGGG
USP14/R	TTTGGCTGAGGGTTCTTCTGG

### Statistical analysis

The differences of variables between each group and within a group in different days were analyzed. *P*-values were calculated by the Mann-Whitney and ANOVA methods using SPSS 16.0 (Chicago, IL, USA), and *P*-Value <0.05 were considered as significant. All graphs were evaluated with Graph Pad Prism 6.0 (Graph Pad Software, Inc., San Diego, CA)

## Results

### HU concentration in cell culture

The results of optimizing HU concentration showed that 100 μM of HU had the best results, minimum toxic effects, and maximum total Hb concentration. In 200 μM and 400 μM concentrations, despite showing positive effects on Hb production, most of the precursor cells died on days 8 and 4, respectively (Fig. 1A, B).

### The results of human erythroid precursor cells culture

**Fig. 2:**
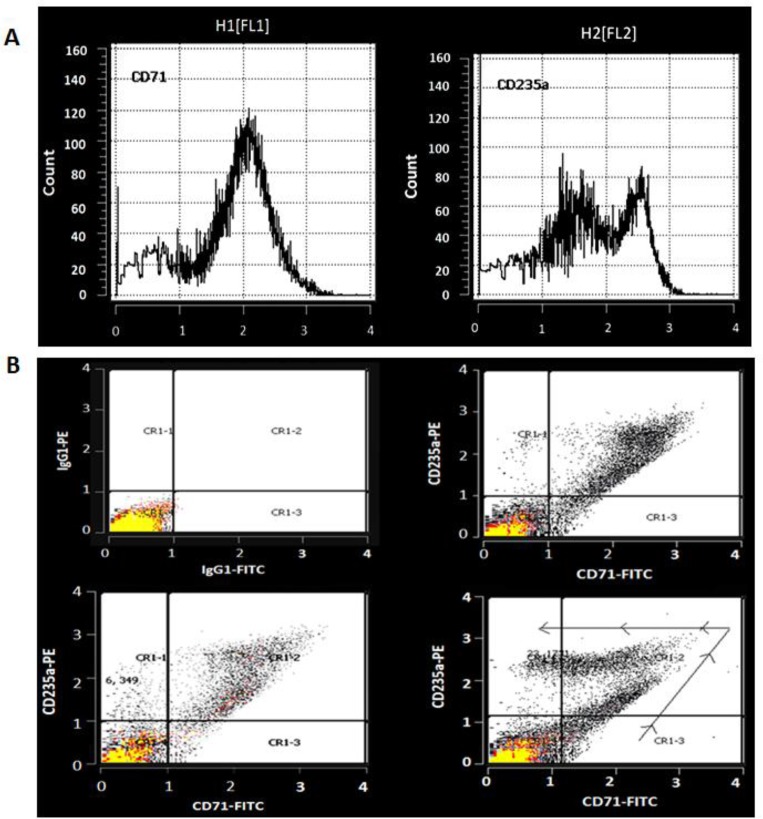
A) Histograms of flow cytometry evaluation of precursor's cell surface markers; The left histogram shows population of precursors expressing CD71 marker. The right histogram shows two distinct populations of precursors expressing CD235a. B) Evaluating precursors, Isotype control (up-left), on day 12 (upright), day 14 (down-left), and day 16, 48 h after HU treatment (downright). The boomerang-like chart shows the presence of a wide range of erythroid precursors, from different part of maturation stage, from the most immature cells (CR1-3) up to mature RBCs (CR1-1)

**Fig. 3:**
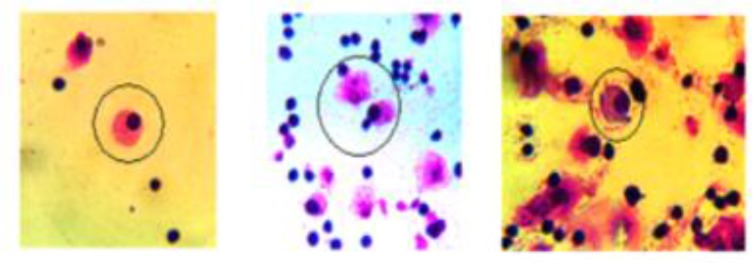
Erythroid precursor cells on day 4 of phase three. A nucleated red blood cell (NRBC) (in the circle) with pyknotic nuclei and pink-red cytoplasm (Left). Enucleating process in NRBC (Middle). An NRBC (in circle) with Violet cytoplasm (Right)

Isolated PBMCs from donor’s peripheral blood were cultured as mentioned in material and methods. The flow cytometry analysis showed that the cells express dual of CD71/CD235a markers on days 12 and 14 the non-day 16, the expression of CD71 was decreased while CD235a expression was increased which these results indicate PBMCs were differentiated to erythroid precursors and maturation of erythroid series (Fig. 2). In addition, the cell morphology in cell culture on day 18 showed late stage of erythroid maturation (Orthochromatic normoblast) (Fig. 3).

### All groups showed increased amount of total Hb after HU exposure

The Hb concentration of R and NR groups were significantly higher than C group in cell culture medium on day 14 respectively (*P*=0.002 and 0.015), while the Hb concentration was not significantly different between R and NR group on day 14 (*P*=0.334). Hb content was significantly increased in all groups after HU treatment on day 16 (Table 2). Hb fold changes after HU exposure were not significantly different between R and NR groups.

**Table 2:** Evaluation of Hb/cell (10^−8^ g/dl) changes in three groups

**HU**		**Hb (day 14, before HU)**	**Hb (day 16, after HU)**	**Difference (day 16 - day 14)**	***P*-value**	**Fold Change**
non-Responder	Mean± SD	17.25± 5.78	24.75± 6.94	7.50	0.045	1.43
Responder	Mean± SD	20.25± 7.46	32.33± 4.93	12.08	0.042	1.60
Control	Mean± SD	9.25± 4.62	14.70± 2.66	5.45	0.048	1.59

### BCL11A and SOX6 expression

**Fig. 4:**
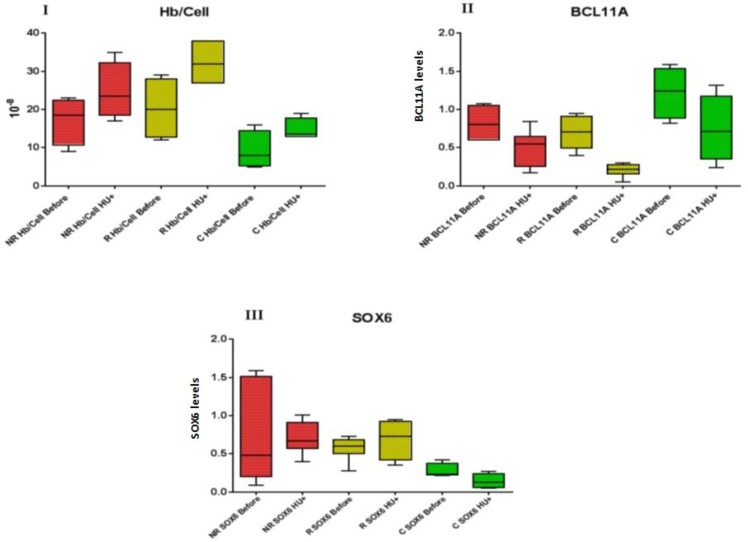
I) Comparison of Hb levels in investigated groups before and after HU treatment, II) Expression of BCL11A before and after HU treatment, III) Expression of SOX6 before and after HU treatment

BCL11A expression was significantly decreased after exposure to HU in all groups (R: *P*=0.000, NR: *P*=0.048, C: *P*=0.000) while SOX6 expression was downregulated in C group (*P*=0.014), and its expression was not significantly changed on R and NR groups after HU treatment (R: *P*=0.402, NR: *P*=0.757). The levels of BCL11A and SOX6 expression are presented in Fig. 4II and 3III.

## Discussion

Among chemical Hb F inducing agents, HU is the only FDA approved compound administered for decreasing clinical complications of sickle cell anemia and β-thalassemia patients. Different studies have reported that HU causes increased HbF levels and ameliorates clinical manifestations of these diseases. However, in BT patients, different clinical response to HU was reported and in the present study, in vitro Hb production between thalassemia patients R and NR to HU was evaluated (4–5).

In the present study, none of the BT patients carried α-gene deletions, they were homozygote for *IVSII-1G>A* mutation, and had the same *XmnI* SNP (+/+) - (the most significant genetic factor associated with high HbF expression located at −158 of ^G^γ-globin gene) (16) in order to rule out the effects of these factors on Hb induction in R and NR groups. Γ-globin expression in R group was higher than NR group after HU treatment (17–20) that indicates positive role of HU on increasing in-vitro γ-globin expression and subsequently increasing total Hb levels.

We have observed lower amount of Hb in healthy group in comparison to two thalassemia groups. The cells isolated from healthy individual are less adapted to the mechanisms confronting with cellular stress; therefore, toxic conditions such as cell culture may increase cell damage or cell cycle arrest. While in BT patients due to toxic agents such as iron free radicals, the cells are more adapted to these stresses than the cells of healthy individuals. Hence, these mentioned processes may have a possible role in decreasing Hb production in healthy group in comparison to BT patient son day 14 of the present study (21, 22).

Response to HU has evaluated in cell cultures of 8R and 8NR BT patients that 69 and 19% of chromosomes carried XmnI SNP in R and NR Patients, respectively (17). They reported that baseline HbF production before HU treatment was higher in R group than NR group while our results showed that Hb concentration per cell on day 14 is not significantly different between R and NR groups. These various results can be related to different genetic background of the patients in two studies. In our study, all of the patients were homozygote for the IVSII-1G>A mutation and XmnI SNP while the frequency of XmnI SNP in R group was higher than NR group. Besides, the patients did not have the same mutation on β-globin gene. Therefore, the higher frequency of XmnI SNP on R group may be responsible for higher HbF production.

In addition to *cis*-elements in β-globin gene cluster, other factors outside of β-globin gene cluster are involved in Hb switching. BCL11A is one of the main proteins responsible for γ-globin gene silencing (23, 24). BCL11A protein directly contacts with the LCR in β-globin gene cluster and repressive sequences located on downstream of β-globin gene and causes LCR shift to β-globin gene promoter and reduction of γ-globin expression (24–26). HU treatment leads to downregulation of BCL11A expression and as result induction of γ-globin expression. In children with SCD, HU caused increased HbF level via decreases of BCL11A expression (27, 28). In this study, with downregulation of BCL11A expression after HU treatment, the total Hb was elevated in all groups.

SOX6 is another important protein involved in γ-globin gene expression that in addition to cooperation in γ-globin gene silencing by helping BCL11A, plays an important role in erythropoiesis progression to the final differentiation (29–30). In erythroid progenitors, SOX6 plays an important role in up-regulation of Bcl-xL (an antiapoptotic gene) at late stages of erythropoiesis (31). Erythropoietin (EPO) primarily induces Bcl-xL expression in early erythropoiesis thus when the effects of EPO on stimulating Bcl-xL expression diminishes, SOX6 acts as compensatory factors to maintain Bcl-xL in order to protect the cells from death (32–34). This mechanism may be responsible for up-regulation of SOX6 in BT patients. Further studies are recommended to clarify the role of SOX6 in apoptosis process and γ-globin expression.

Individual differences between patients can affect response to HU and Hb production in patients (35–37).

## Conclusion

Regardless of clinical responsiveness to HU, this drug was able to increase production of Hb in NR group similar to other groups. Therefore, increased amount of total Hb on cell culture cannot predict clinical response to HU, because different mechanisms including intracellular factors.

## Ethical considerations

Ethical issues (Including plagiarism, informed consent, misconduct, data fabrication and/or falsification, double publication and/or submission, redundancy, etc.) have been completely observed by the authors.
